# Plastics shape the black soldier fly larvae gut microbiome and select for biodegrading functions

**DOI:** 10.1186/s40168-023-01649-0

**Published:** 2023-09-14

**Authors:** Francesca De Filippis, Marco Bonelli, Daniele Bruno, Giuseppina Sequino, Aurora Montali, Marcella Reguzzoni, Edoardo Pasolli, Davide Savy, Silvana Cangemi, Vincenza Cozzolino, Gianluca Tettamanti, Danilo Ercolini, Morena Casartelli, Silvia Caccia

**Affiliations:** 1 Department of Agricultural Sciences, University of Naples Federico II, Portici, Italy; 2 Task Force on Microbiome Studies, University of Naples Federico II, Naples, Italy; 3 Department of Biosciences, University of Milan, Milan, Italy; 4 Department of Biotechnology and Life Sciences, University of Insubria, Varese, Italy; 5 Department of Medicine and Surgery, University of Insubria, Varese, Italy; 6 Interdepartmental Research Centre of Nuclear Magnetic Resonance for the Environment, Agri-Food and New Materials (CERMANU), University of Naples Federico II, Portici, Italy; 7 Interuniversity Center for Studies on Bioinspired Agro-Environmental Technology (BAT Center), University of Naples Federico II, Portici, Italy

**Keywords:** Insect gut microbiota, Insect gut microbiome, Plastic biodegradation, *Hermetia illucens*, Bioconversion

## Abstract

**Background:**

In the last few years, considerable attention has been focused on the plastic-degrading capability of insects and their gut microbiota in order to develop novel, effective, and green strategies for plastic waste management. Although many analyses based on *16S rRNA* gene sequencing are available, an in-depth analysis of the insect gut microbiome to identify genes with plastic-degrading potential is still lacking.

**Results:**

In the present work, we aim to fill this gap using Black Soldier Fly (BSF) as insect model. BSF larvae have proven capability to efficiently bioconvert a wide variety of organic wastes but, surprisingly, have never been considered for plastic degradation. BSF larvae were reared on two widely used plastic polymers and shotgun metagenomics was exploited to evaluate if and how plastic-containing diets affect composition and functions of the gut microbial community. The high-definition picture of the BSF gut microbiome gave access for the first time to the genomes of culturable and unculturable microorganisms in the gut of insects reared on plastics and revealed that (i) plastics significantly shaped bacterial composition at species and strain level, and (ii) functions that trigger the degradation of the polymer chains, i.e., DyP-type peroxidases, multicopper oxidases, and alkane monooxygenases, were highly enriched in the metagenomes upon exposure to plastics, consistently with the evidences obtained by scanning electron microscopy and ^1^H nuclear magnetic resonance analyses on plastics.

**Conclusions:**

In addition to highlighting that the astonishing plasticity of the microbiota composition of BSF larvae is associated with functional shifts in the insect microbiome, the present work sets the stage for exploiting BSF larvae as “bioincubators” to isolate microbial strains and enzymes for the development of innovative plastic biodegradation strategies. However, most importantly, the larvae constitute a source of enzymes to be evolved and valorized by pioneering synthetic biology approaches.

Video Abstract

**Supplementary Information:**

The online version contains supplementary material available at 10.1186/s40168-023-01649-0.

## Background

Accumulation of plastics in the environment that are fragmentated into micro- and nanoparticles by biotic and abiotic processes represents a global concern since as it has a dramatic impact on ecosystems [1–5]. Therefore, it is crucial that traditional synthetic plastics are replaced by biodegradable and bio-based polymers [6, 7] and that the sustainability of plastic products is improved by developing integrated strategies to reuse, recycle, and recover petroleum-based plastics [8]. Unfortunately, efficient and eco-friendly methods to dispose or recycle plastics and plastic-containing wastes are still unsatisfactory [9–11]. However, innovative and challenging solutions for biological recycling of plastics have recently been proposed to ensure a more bio-based, low-impact, and circular management of these polymers. In this context, using microbial enzymes to degrade plastics has been widely explored [5, 8, 12, 13]. Microbes producing plastic-degrading enzymes have been isolated from the most disparate sources, such as marine ecosystems, soil, plastic landfills, and invertebrates [13–19]. Although some insects are able to chew plastics from food packaging [20–22], their potential to degrade ingested plastics has only been described in 2014 for *Plodia interpunctella* larvae [23]. Since that report, a polyethylene (PE)- or polystyrene (PS)-degrading capacity was reported in several insect larvae and a few bacterial strains with PE- or PS-degrading activity have been isolated (e.g., [23–27]). It is worth mentioning that the vast majority of these studies concerned the greater waxworm *Galleria mellonella*, the mealworm *Tenebrio molitor* and the superworm *Zophobas atratus*. While the eating habits of waxworms (i.e., they feed on beeswax, which is composed of hydrocarbons as alkanes and alkenes) endow these insects with the ability to digest and derive energy from plastics [28–33], evidence indicates that gut microbiota plays an essential role in degrading plastics in other insects (e.g., [25, 34–39]), with a few exceptions [40]. Nevertheless, an in-depth characterization of the microbial community in insects reared on plastics at a compositional (i.e., whether plastics are able to shape the microbiota at species and strain level) and genomic (i.e., whether and how microbial genomes are enriched in plastic-degradation functions) level is still lacking.

The larvae of Black Soldier Fly (BSF), *Hermetia illucens* (Diptera: Stratiomyidae), are able to grow on a wide variety of organic substrates, including side streams and wastes, and their gut microbiota is significantly shaped by the composition of the rearing substrate [41–45]. The close relationship between diet and gut microbiota, which contributes to host fitness, for instance, by supplying essential nutrients and facilitating food digestion [46–48], gives rise to the intriguing prospect of finely shaping the microbiota by manipulating the diet to select specific biological functions. Despite the fact that the extraordinary feeding plasticity and the high bioconversion capability of BSF larvae are increasingly gaining attention [49–53], the possibility of exploiting this insect for degrading and/or bioconverting plastic waste, supported by the selection of specific plastic-degrading functions in its microbiome has never been explored.

The aim of the present study was to investigate whether the microbiome of BSF larvae could be a source of plastic-degrading functions. Our results indicate that BSF larvae fed on PE- or PS-based rearing substrates show an astonishing dynamism as holobionts as the ingestion of plastics significantly shapes the composition of their gut microbiota at the species and strain level, and the microbiome is enriched in specific plastic-degrading functions. Notably, metagenomics evidence is supported by both ultrastructural polymer alterations and ^1^H nuclear magnetic resonance (^1^H NMR) analysis, which confirm the actual degradation of plastics. This study, considering for the first time genomic information about both culturable and unculturable microorganisms, emphasizes the effectiveness of BSF larvae not only in bioconversion processes, as already demonstrated for many byproducts and organic wastes, but also as a rich source of genes coding for enzymes with a polymer-degrading capability that can be evolved and improved to open new scenarios for developing innovative, effective, and green bio-tools for plastic disposal and recycling.

## Methods

### Insect rearing

BSF eggs were collected from a colony established in 2015 at the University of Insubria (Varese, Italy) and maintained as reported in [42]. Briefly, the eggs were laid on a Petri dish (9 × 1.5 cm) with a standard diet (STDd) widely used for fly larvae rearing [54], composed of wheat bran (50%), alfalfa meal (20%), and corn meal (30%) mixed in the ratio 1:1 dry matter/water. Eggs were maintained in a humid chamber at 27 °C until hatching, and after emergence, BSF larvae were reared on STDd for 4 days in the presence of nipagin (methyl 4-hydroxybenzoate) to avoid mold growth (a 18% w/v stock solution in absolute ethanol was prepared; 1 ml of a 1.7% v/v dilution in water of the stock solution was added to each gram of STDd). The larvae were then placed in plastic boxes (16 × 16 × 9 cm) containing STDd without nipagin until the 10th day after hatching (defined as time zero, *T*_zero_), at which time larvae (approximate weight of 70 mg) were transferred to the experimental rearing substrates (i.e., AGARd, PEd, and PSd, where “d” stands for diet) for 2 weeks, while control larvae were maintained on STDd until 25% of insects reached pupal stage. Briefly, agar-based diets containing PE (particles major axis between 400 and 1000 µm) or PS (particles major axis between 400 and 800 µm) powder (Powderex, Italy) were prepared by dissolving 4% (w/v) agar powder (Merck, DE) in hot (95 °C) distilled water; then, the solution was cooled to 60 °C, and 20% (w/v) of the polymer was added to the agar solution. When the suspension reached room temperature and solidified, the diet was mechanically mashed. AGARd was prepared with the same procedure without adding polymers. Before transferring the larvae to the experimental rearing substrates, they were kept on STDd for 6 days after the weaning period as preliminary experiments showed that once moved to AGARd, PEd, and PSd they did not gain weight (Additional file 1: Figure S1). This procedure allowed larvae to reach dimensions ensuring that proper amounts of DNA could be recovered from midgut content for metagenomics analysis.

Batches of 150 larvae were placed in a plastic box (14 × 14 × 7.5 cm), and fed ad libitum with STDd, AGARd, PEd, and PSd. At least 3 independent groups of 150 larvae, derived from eggs laid by females of different generations, were set up for each diet. The larvae were maintained at 27.0 ± 0.5 °C, 70 ± 5% relative humidity, in the dark. For STDd, fresh diet was added every 4 or 5 days until larvae reached the last instar; samples of 30 randomly selected individuals were weighed every 2–4 days until 25% of insects reached the pupal stage. Samples of 30 randomly selected larvae reared on AGARd, PEd, and PSd were weighed 7 and 14 days after the beginning of rearing on the experimental substrates. Before weighing, larvae were washed in tap water to remove substrate residues and then wiped dry.

### Scanning electron microscopy analysis

After rearing BSF larvae on PEd and PSd for 2 weeks, the substrates were collected to evaluate any structural alterations on the surface of the plastic particles (post-rearing samples). PEd and PSd kept in the same conditions without the larvae served as controls. All diet samples were stored at − 20 °C until use. Moreover, nonagarized PS and PE particles (i.e., plastic powders) were analyzed, too. After thawing, samples were placed in cell strainers (40-μm mesh size) (BD Biosciences, Milan, Italy) and extensively washed with 100 mM NaCl and Milli-Q water. Samples were then mounted on stubs, gold-coated with a Sputter K250 coater, and finally analyzed with a Scanning Electron Microscope (SEM)-FEG XL-30 microscope (Philips, Eindhoven, The Netherlands) (Centro Grandi Attrezzature, University of Insubria). For each condition, the analysis was performed in triplicate.

### Metagenomic analyses of the midgut content of BSF larvae

**Fig. 1:**
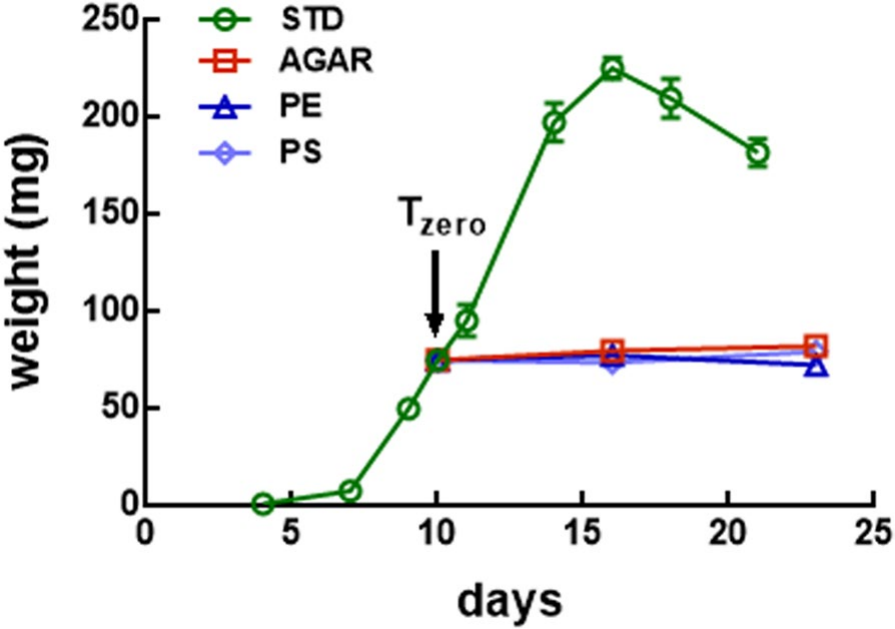
Growth curves of BSF larvae reared on standard diet (STD samples), agar-based substrates containing polyethylene (PE) and polystryrene (PS), and agar (PE, PS, and AGAR samples, respectively). After hatching, larvae were reared on standard diet or after 10 days (*T*_zero_) were moved from this diet to agar-based substrates containing PE or PS (or moistured agar that served as control) for 2 weeks

DNA was extracted from the midgut content of larvae at different time points (*T*_zero_, after 2 weeks for larvae reared on AGARd, PEd, and PSd, and on actively feeding last instar larvae reared on STDd; see legend to Fig. 1 for details). Analysis was performed on this region of the gut as the midgut is the main site of digestion and microorganisms present in the lumen of this tract can significantly contribute to exploitation of the rearing substrates. Before DNA extraction, larvae were washed with 70% ethanol in distilled water and then dissected under a stereomicroscope using sterile tweezers and scissors to avoid cross-contamination of the samples. For the dissection of each larva, a new Petri dish was used, and tweezers and scissors were washed with 70% ethanol. The midgut was isolated in sterile phosphate-buffered saline (137 mM NaCl, 2.7 mM KCl, 4.3 mM Na_2_HPO_4_, and 1.4 mM KH_2_PO_4_; pH 7.4) in a sterile Petri dish (5.5 × 1.3 cm). The midgut content enclosed in the peritrophic matrix (PM) was isolated from the midgut and immediately frozen in a 1.5-ml tube in dry ice. For each diet and time point, samples (each sample is made of pools of 15–30 PMs) were collected and stored at − 80 °C until DNA extraction. Sample numbers (at least 12 and 6 samples for *16S rRNA* sequencing and shotgun metagenomics, respectively, for each rearing substrate) guaranteed the quality of the sequencing data obtained. The exact number of samples for each experiment and conditions are reported in figures and legends for *16S rRNA* sequencing and in Additional file 2: Table S1 for shotgun metagenomics.

DNA was extracted following the Standard Operating Procedures developed by the International Human Microbiome Standard Consortium (SOP_07; www.microbiome-standards.org). Total DNA was purified using the NucleoSpin gDNA Clean up kit (Macherey–Nagel, Dueren, Germany) and quantified by Qubit fluorometer using the Qubit dsDNA High-Sensitivity Assay kit (Thermo Fisher Scientific, MA, USA).

The V3–V4 region of the *16S rRNA* gene was amplified using primers S-D-Bact-0341F5’- CCTACGGGNGGCWGCAG and S-D-Bact-0785R5’-GACTACHVGGGTATCTAATCC [55] as described previously [56]. Briefly, the following PCR conditions were used: an initial denaturation at 95 °C for 3 min, followed by 25 cycles of 95 °C for 30 s, 55 °C for 30 s, 72 °C for 30 s, and a final extension at 72 °C for 5 min.

Amplicon libraries were sequenced by Novogene (Cambridge, UK) on a MiSeq platform, leading to 2 × 250 bp, paired-end reads. For shotgun metagenome sequencing, a subset of microbial DNA samples used for *16S rRNA* analysis were randomly chosen (6 *T*_zero_, 6 STD, 18 AGAR, 14 PE and 20 PS samples, each obtained from pooling 15–30 PMs). DNA libraries were sequenced on Illumina NovaSeq platform, generating to 2 × 150 bp, paired-end reads. Host reads contamination was removed mapping reads to the *H. illucens* genome (NCBI Accession Number: PRJEB37575) by using the Best Match Tagger (BMTagger; https://www.westgrid.ca/support/software/bmtagger). All raw data are available in Additional files or on NCBI with the accession numbers provided in “Availability of data and materials.”

### Bioinformatics analyses

Demultiplexed forward and reverse *16S rRNA* gene reads were joined by using FLASH [57]. Reads with a Phred score < 20 were trimmed by PRINSEQ 0.20.4 [58] and those shorter than 300 bp were discarded. High-quality reads were analyzed by QIIME 1.9 [59], with a pipeline described previously [56]. Briefly, operational taxonomic units (OTU) were picked at 97% of identity using a de novo approach and the uclust method, and taxonomic assignments were obtained by using the RDP classifier and the Greengenes database [60]. OTU tables were rarefied to the lowest number of sequences per sample.

Shotgun metagenomics reads were quality-filtered using PRINSEQ 0.20.4: reads with bases having a Phred score < 20 were trimmed and those < 75 bp were discarded. High-quality reads were imported in mOTUs2 [61] to obtain species-level, quantitative taxonomic profiles. The standard mOTUs database was used for taxonomic assignment.

High-quality reads were assembled independently using MEGAHIT v. 1.2.2 ([62]; options: -m 0.85 –min-contig-len 1000 –k-list 21,33,55,71,81,91) and contigs > 1000 bp were used to predict genes by using MetaGeneMark v. 3.26 [63]. Assembly results are reported in Additional file 2: Table S1. Predicted genes were aligned (using DIAMOND v. 2.0.4 and the option –very-sensitive; [64]) against a custom database including known microbial genes potentially involved in plastic degradation (see “Custom database preparation”). An e-value cutoff of 1e − 5 was applied, and a hit was required to display > 95% of identity over at least 50% of the query length. To obtain the gene abundance, short reads were mapped to the genes using Bowtie2 (options: –very-sensitive-local –no-unal; [65]) and the number of mapped reads was normalized using the RPKM method (reads per kilobase per million mapped reads) and considering the formula (number of hits for gene *a*/gene *a* length)/total number of mapped reads per sample) as reported by [66].

In order to obtain contig coverage, reads of each sample were also mapped against the contigs reconstructed for that sample, using Bowtie2 (options: –very-sensitive-local –no-unal). Contigs (> 1500 bp) were binned using MetaBAT2 v. 2.12.1 with standard parameters [67], and the quality of Metagenome-Assembled Genomes (MAGs) was estimated with CheckM v. 1.1.3 [68]. Only MAGs with > 50% completeness and < 5% contamination were retained for further analyses. MAGs binned in this study were clustered to a genomic database including 107,442 high-quality MAGs previously reconstructed from human metagenomes [69] and 185,939 genomes from isolates downloaded from NCBI RefSeq on May 2020. Pairwise genetic distances between genomes were calculated using Mash (version 2.0; option ‘‘-s 10,000’’ for sketching; [70]). A Mash distance < 5% from any of the database genomes was considered to place the MAG within the relative species-level genome bin (SGB). When a MAG showed > 5% distance from any reference genomes, it was considered a novel species (unknown SGB, uSGB). In this case, the taxonomic assignment was made at genus (> 5% and < 15% distance), family (> 15% and < 25% distance), or phylum (> 25% distance) level. These thresholds were used accordingly to [69]. To define subspecies, pairwise average nucleotide identities (ANI) were estimated between genomes falling in the same SGB through FastANI [71] and clustered using the Partitioning Around Medoid (PAM) algorithm in R, as described recently [72]. The number of subspecies was estimated by identifying the optimal number of clusters for each SGB, using the *prediction.strength* function in R package fpc. We required the number of clusters to be supported by a prediction strength ≥ 0.8. RAxML 8.0 [73] was used to generate species-specific phylogenetic trees, which were visualized in iTOL v. 5.5.1 [74].

### Custom database preparation

To prepare a database for aligning of the genes predicted from shotgun metagenomics, preliminary bibliographic research was carried out to individuate target proteins/genes involved or putatively involved in plastic polymer degradation. These targets (i.e., laccase, DyP-type peroxidase, lignin peroxidase, manganese peroxidase, versatile peroxidase, alkane hydroxylase, and alkane monooxygenase) were used as keywords to mine available databases (NCBI, https://lcced.biocatnet.de/) and the information was manually refined for duplicates. A detailed file with gene or protein names and accession numbers is reported in Additional file 3: Table S2.

### Proton nuclear magnetic resonance analysis

To evaluate changes due to degradation processes in PE after BSF larvae rearing, ^1^H NMR analysis was carried out. Post-rearing PEd (i.e., PE-based rearing substrate where larvae were reared for 2 weeks), PEd (PE-based rearing substrate kept in the same rearing conditions for 2 weeks without the larvae), and the relative controls (i.e., AGAR-based rearing substrate where larvae were reared for 2 weeks, and AGAR-based diet kept in the same rearing conditions for 2 weeks without the larvae) were analyzed. All the samples were freeze-dried. Before conducting the liquid-state ^1^H NMR analysis, samples (30 mg) were extracted with 2 ml of deuterated-chloroform. The samples were sonicated (30 min) and filtered through 0.22-μm polyvinylidene fluoride filters before loading into an NMR tube. ^1^H NMR spectra were acquired at 25 ± 1 °C, with tetramethylsilane as an internal standard, through a 9.4-Tesla Bruker Avance Magnet (Bruker Biospin, Rheinstetten, Germany) equipped with a BBI probe operating at the resonance frequencies of 400 MHz for ^1^H. Spectra were analyzed using MestReNova software. ^1^H NMR spectra were processed by doubling the number of data points to increase the spectral digital resolution (zero-filling). Furthermore, the signal-to-noise ratio was enhanced by applying a Lorentzian weighing function with a line broadening equal to 0.60 Hz (apodization). The transformed spectrum was therefore phased (zero- and first-order phase correction) and the baseline adjusted. Finally, the chemical shift of the internal standard was set to zero.

### Statistical analysis

Differences in the overall microbiome taxonomic composition according to diet were assessed by PERMANOVA (Permutational Multivariate Analysis of Variance, *adonis* function, vegan R package) computed on Jaccard distance matrix (*p* < 0.05). Alpha diversity indices were computed on mOTUs species-level table by using the *vegdist* function (vegan R package). Comparisons of taxa, alpha-diversity indices, or gene abundance between groups were carried out using pairwise Wilcoxon tests and results were considered significant at *p* < 0.05. Classical multidimensional scaling (MDS, *cmdscale* function, stats R package) was carried out on ANI distance matrices.

## Results

### BSF larvae develop on plastic-based diets

BSF larvae were reared on agarized diets containing PE or PS (agar alone served as control diet). The consistence of the agarized substrates allowed the larvae to burrow and move into the diet, which are important features for their gregarious habits [75]. As preliminary experiments showed that BSF larvae were able to survive and complete their development on diets containing agarized plastics only (Additional file 1: Figure S1) and the aim of this study was to select a plastic-degrading microbiome rather than to exploit the insects’ ability to reduce and bioconvert plastic waste, no additional nutrients were added to the substrate. BSF larvae were reared on standard diet (STDd) or moved from STDd to agarized substrates containing PE (PEd) or PS (PSd), or on agar alone (AGARd, used as control) after 10 days (*T*_zero_). Compared to larvae reared on STDd that developed and all pupated within about 20 days, larvae moved to PEd, PSd, and AGARd did not gain weight (Fig. 1) and reached the pupal stage about 10–15 days later (Additional file 1: Figure S1), as has consistently been shown in other species [24, 25, 34, 38, 76].

### Clear structural alterations are present on PS and PE surfaces after BSF larvae rearing

**Fig. 2:**
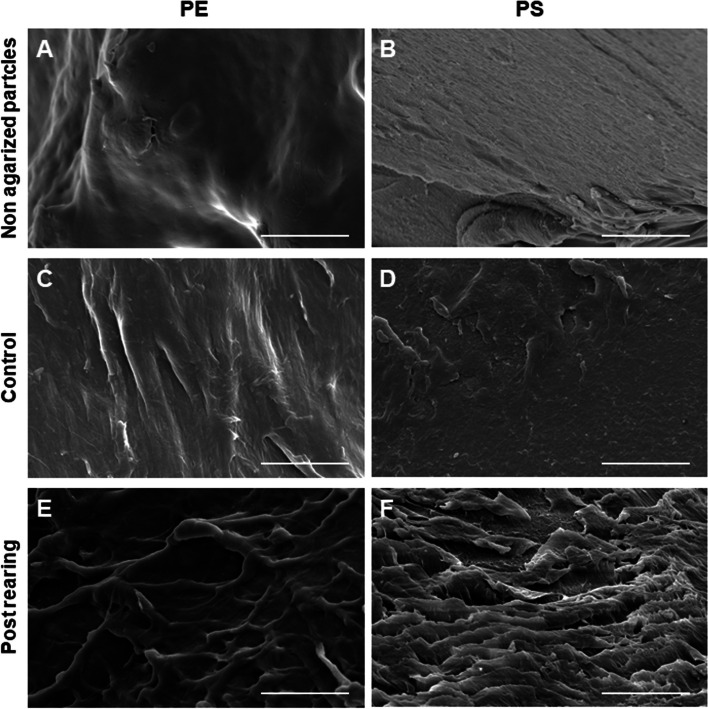
Scanning electron microscopy analysis. Morphology of masterbatch polyethylene (PE) and polystyrene (PS) particles used to prepare the rearing substrates (non agarized particles) (**A**, **B**). Morphology of plastics in the rearing substrate without the larvae (control) (**C**, **D**) and after rearing the larvae for 2 weeks (post-rearing) (**E**, **F**). Bars: 5 μm

SEM analysis was performed to evaluate alterations on the surface of plastic particles after BSF larvae rearing. The surface of PS and PE particles used to prepare the rearing diets appeared mostly smooth (Fig. 2A, B). The same features were observed in control samples (i.e., PS- and PE-based rearing substrates kept for 2 weeks without the larvae) (Fig. 2C, D; Additional file 1: Figure S2). In contrast, micro- and nanoscale cavities and pits on the surfaces were visible in post-rearing samples (Fig. 2E, F; Additional file 1: Figure S2). The hardness of the material and the size of the surface alterations (less than 1 µm) suggest that structural alterations occurred in the gut lumen, rather than by mechanical damage due to insect mouthparts.

### Rearing BSF larvae on plastic-based diets significantly and deeply shapes their gut microbiota

**Fig. 3:**
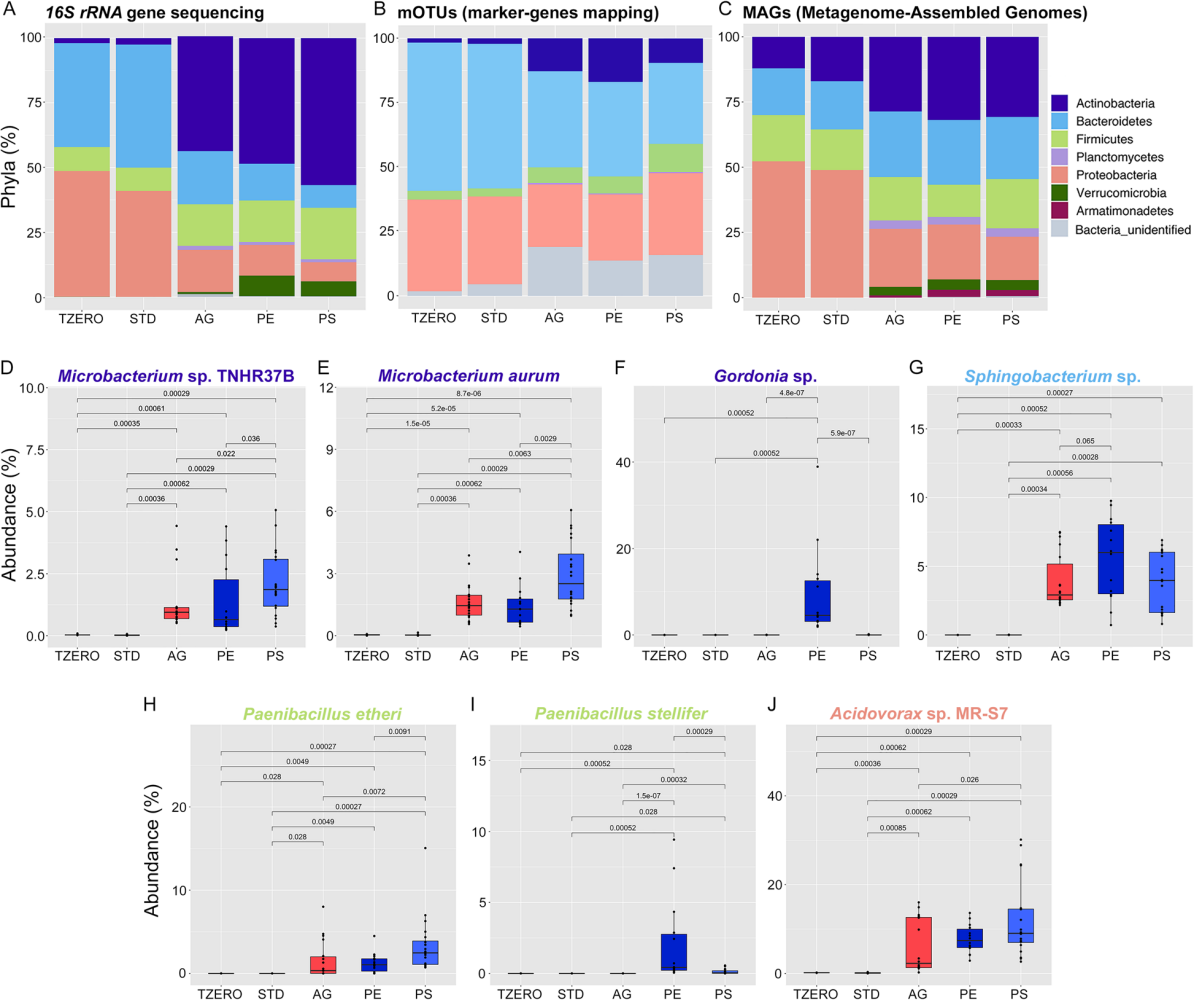
Midgut microbiota and microbiome composition of BSF larvae reared on standard diet (STDd), agar or agar-based substrates containing polyethylene (PE) or polystyrene (PS). Midgut content was isolated from larvae reared on standard diet for 10 days (*T*_zero_ samples) and from larvae that were then moved to PEd or PSd (PE and PS samples, respectively), or AGARd (AGAR samples) for 2 weeks or left on STDd (STD samples) for 4 days from *T*_zero_ (i.e., when the larvae reached the last instar and were still actively feeding). Taxonomic composition at phylum level (% relative abundance) obtained by *16S rRNA* gene sequencing (**A**), mapping metagenomics reads against marker genes through mOTUs (**B**), and assigning taxonomy to metagenome-assembled genomes (MAGs; **C**). Boxplots of major changes in the abundance of species belonging to the most represented phyla determined by shotgun metagenomics read-mapping, are also shown (**D**–**J**)

**Fig. 4:**
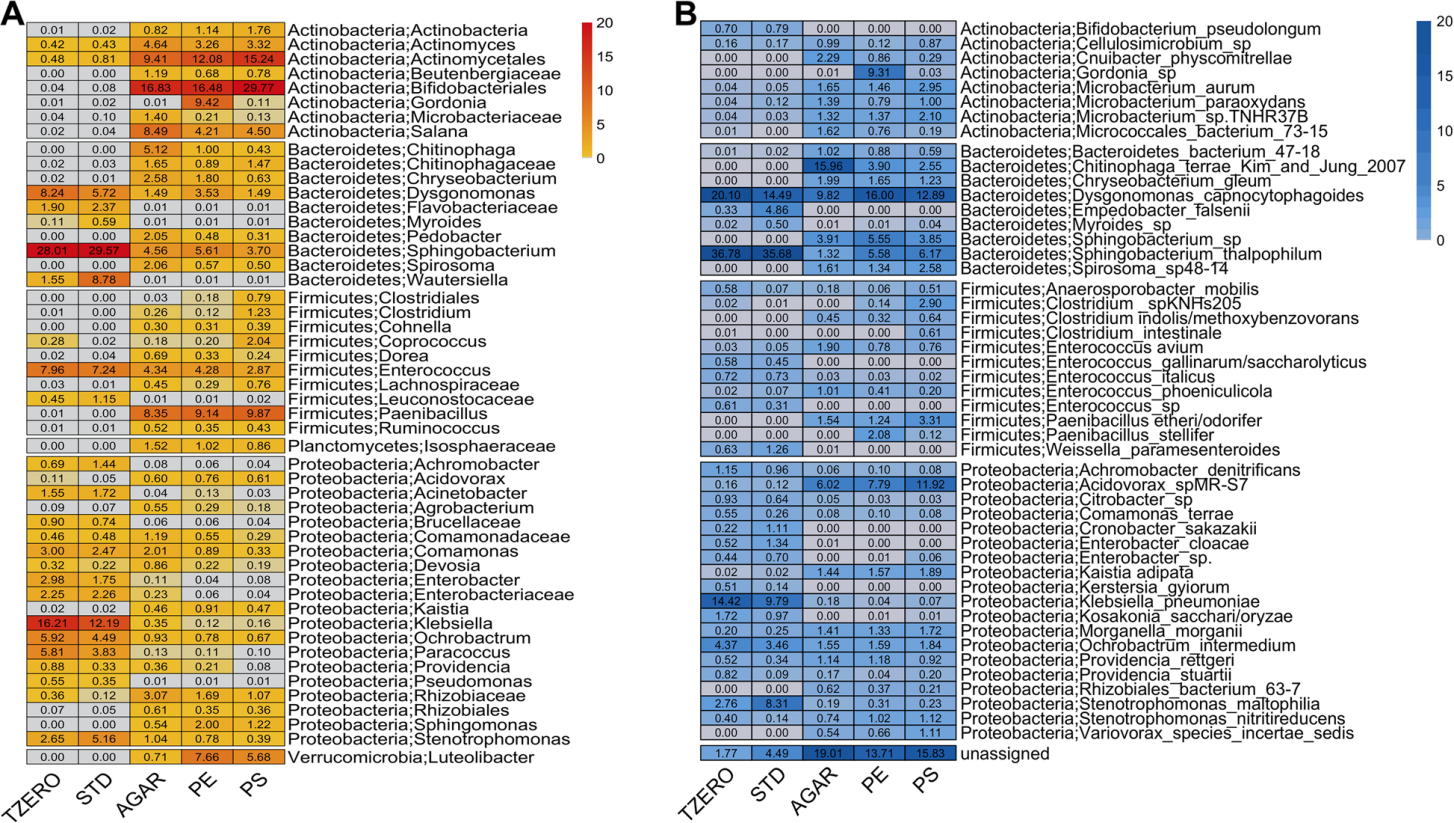
Microbiome composition of *T*_zero_, STD, AGAR, PE, and PS samples determined by *16S rRNA* gene sequencing (**A**) and shotgun metagenomes taxonomic profiling (**B**). Heatplots show the average relative abundance (%) of microbial taxa (OTUs collapsed at genus level or above; (**A**) or species (**B**) with an average relative abundance > 0.5% in at least one group

A preliminary analysis was performed to offer a picture of the microbiota composition in plastic-ingesting BSF larvae using *16S rRNA* gene sequencing, which is the most widely used sequencing approach. In only 2 weeks, plastic-containing substrates significantly affected the composition of the gut bacterial community compared to larvae maintained on STDd, both at *T*_zero_ and afterwards (referred to as “*T*_zero_” and “STD” samples, respectively) (Figs. 3A and 4A, Additional file 1: Figure S3), as assessed by PERMANOVA computed on Jaccard distance matrix (*p* < 0.05). Considering the taxonomic composition at the phylum level (Fig. 3A), Actinobacteria, which were poorly represented in the microbiota of BSF larvae reared on STDd, became dominant in larvae reared on AGARd, PEd, and PSd (referred as “AGAR”, “PE”, and “PS” samples, respectively) (*p* < 0.05). In particular, Actinomycetales and Bifidobacteriales orders accounted for up to 50% of the midgut microbiota of BSF larvae reared on plastics (Fig. 4A). Actinobacteria species are known to survive during exposure to adverse environmental conditions and to produce enzymes with a broad substrate specificity that confer the ability to these microorganisms to degrade both natural and synthetic complex substances that cannot be easily transformed by other mechanisms [77, 78]. In addition, a relevant change was observed for Verrucomicrobia, which were absent in larvae reared on STDd (*T*_zero_ and STD samples), and reached 7.9 and 5.8% in PE and PS samples, respectively (Additional file 1: Figure S3, Fig. 3A; *p* < 0.05). This phylum was almost exclusively represented by *Luteolibacter*, a genus comprising, to the best of our knowledge, only 10 described species [79] (Fig. 4A). Interestingly, BSF larvae reared on PE and PS were characterized by a relatively complex gut microbiota (Figs. 3A, 4A, Additional file 1: Figure S3) compared to caterpillars (e.g., *G. mellonella*, and *P. interpunctella*) and beetle larvae (e.g., *T. molitor* and *Z. atratus*) that showed the dominance of Firmicutes and Proteobacteria (e.g., [23, 26, 80, 81]).

Taxonomic profiling of shotgun metagenomes by a mapping-based approach was also performed. The profiling showed that bacterial diversity in AGAR, PE, and PS samples significantly increased compared to larvae reared on STDd (Additional file 1: Figure S4). A taxonomic profiling of shotgun metagenomes by a mapping-based approach was also performed. The profiling showed that bacterial diversity in AGAR, PE, and PS samples significantly increased compared to larvae reared on STDd (Additional file 1: Figure S4). This may be linked to the strongly nutrient-deprived environment present in the gut lumen of larvae reared on AGARd, PEd, and PSd. Indeed, nutritional starvation may pose a challenge to the gut microbes, thus favoring extensive interspecies communication and metabolic networking and the establishment of a more complex community [82–84]. The phylum-level abundances of control samples almost mirrored those obtained by *16S rRNA* sequencing (Fig. 3A, B). At species level, some Actinobacteria, *Microbacterium* spp., which were almost absent in controls and present in AGAR-, PE-, and PS-fed larvae (Figs. 3D, E and 4B), have the documented ability to biodegrade complex polymers (e.g., hydrocarbons, cellulose, hemicellulose, lignin, and chitin) [85–87]. Moreover, among Actinobacteria, it is worth noting that a *Gordonia* sp. increased up to about 10% only in insects reared on PE, indicating that it may be involved in degrading this polymer (Figs. 3F and 4B). This evidence is intriguing as *Gordonia* spp. are able to catalyze biotransformation and biodegradation of recalcitrant substances, including hydrocarbons and plasticizers [88–94]. A significant increase of a *Sphingobacterium* sp. (Bacteroidetes) in BSF larvae reared on plastics was observed (i.e., absent in controls and increased to up to 4–5% of the microbiota) (Figs. 3G and 4B). Among other phyla, abundance of *Paenibacillus* spp. (Firmicutes) and *Acidovorax* sp. MR-S7 (Proteobacteria) increased with plastic consumption, particularly in PS (Figs. 3H–J and 4B). Interestingly, some *Acidovorax* strains have been described as effective biodegraders of aromatic compounds, such as cyclic and polycyclic hydrocarbons [95–98], while *Paenibacillus*, previously isolated from hydrocarbon-contaminated soils [99], could have a high biotechnological potential owing to its cellulolytic and ligninolytic activity [100–102] and for producing enzymes involved in PE degradation [16, 103–105].

### The identification by functional metagenomics of genes putatively involved in the degradation of plastics is supported by chemical evidence

A major factor rendering the microbial biodegradation of petroleum-based plastics difficult is their high hydrophobicity which hampers microbial cell adhesion and the effective activity of the secreted hydrophilic enzymes [12, 106]. For this reason, the first and most important step in plastic biodegradation is oxidizing the polymer, which leads to the formation of hydrophilic groups (e.g., hydroxyl and carbonyl groups), enhancing microbial degradation performance and breakage of the polymer. Then, bacteria can internalize and metabolize these degradation products [17, 107, 108]. Several microbial enzymes were reported to mediate the first step of plastic biodegradation, mostly peroxidases (i.e., DyP-type, manganese, lignin, and versatile peroxidases), multicopper oxidases (i.e., laccases), and alkane monooxygenases as alkane hydroxylases [12, 17, 106, 109]. Thus, metagenomes were mined to search for those genes that are potentially involved in upstream PE and PS degradation.

**Fig. 5:**
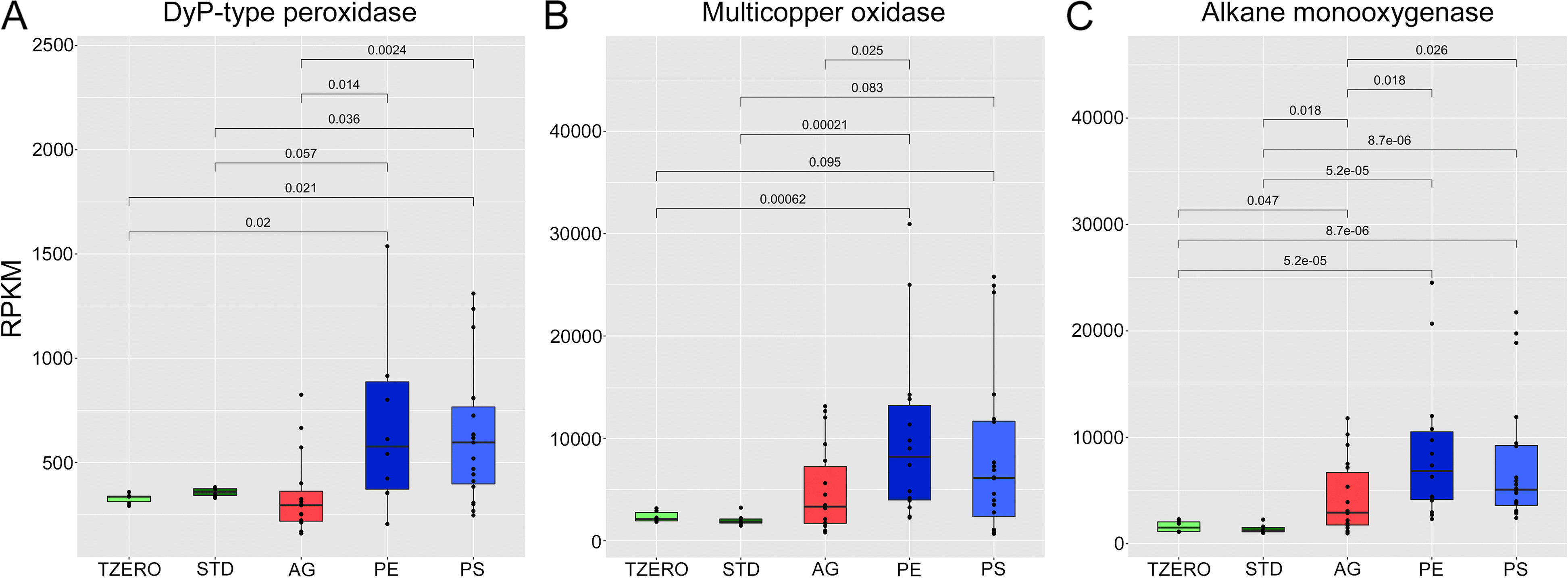
Boxplots showing abundance (expressed as reads per kilobase per million mapped reads, RPKM) of sequences related to genes encoding for DyP-type peroxidases (**A**), multicopper oxidases (**B**), alkane monooxygenase (**C**) in *T*_zero_, standard (STD), AGAR, polyethylene (PE), and polystyrene (PS) samples. Significance was determined by pairwise Wilcoxon tests. Only *p*-values < 0.1 are reported

Genes encoding DyP-type peroxidases were significantly enriched in PE and PS compared to *T*_zero_, STD, and AGAR samples (Fig. 5A), and those encoding multicopper oxidases were significantly more abundant in PE than in other samples (Fig. 5B). Enrichment was also observed for alkane monooxygenase sequences, which increased in both PE and PS samples compared to all the other groups (Fig. 5C). According to the results, the most significant changes were associated with PE ingestion.

**Fig. 6:**
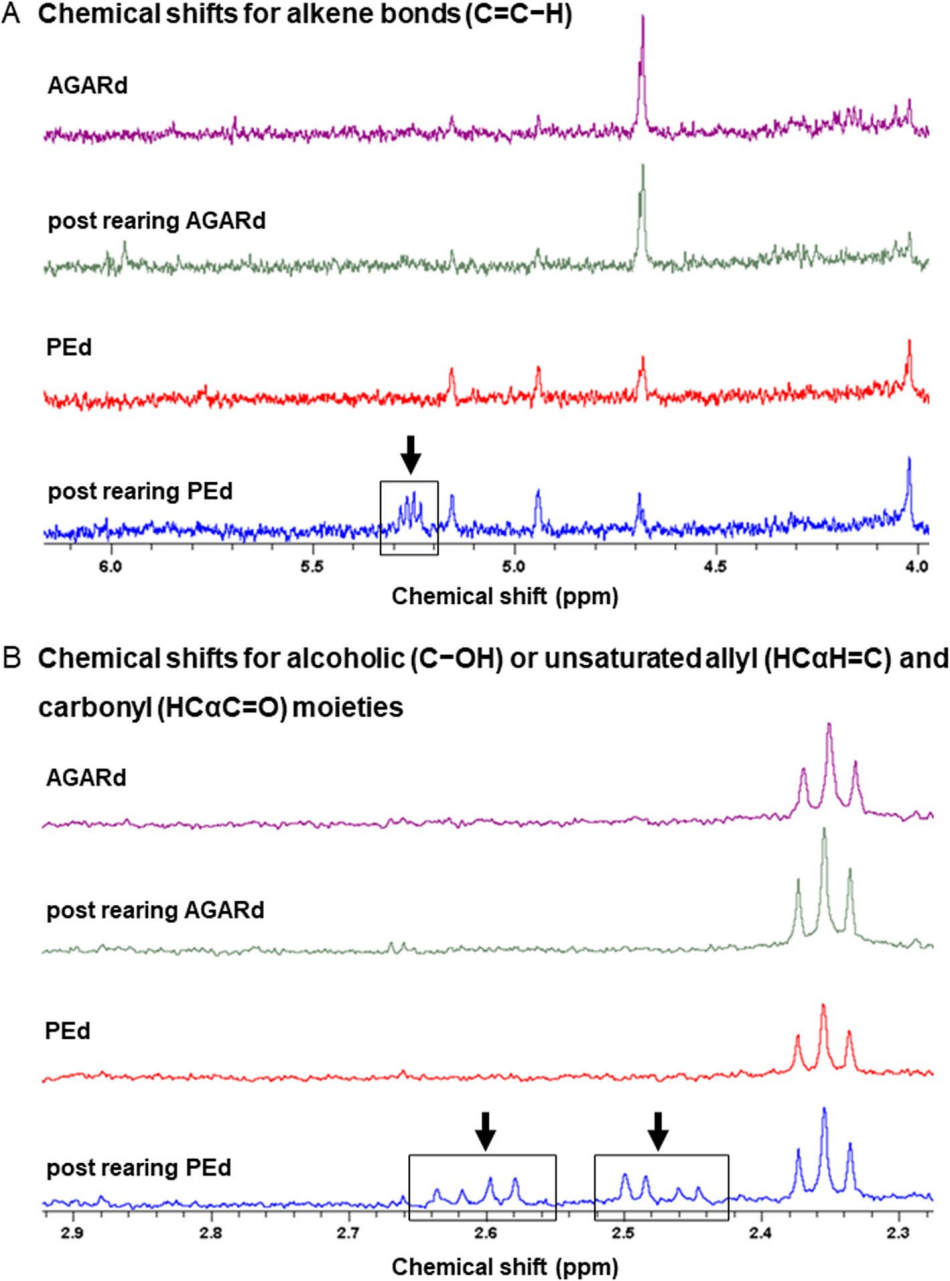
Chemical analysis.^1^H NMR spectra of controls (i.e., AGARd, post-rearing AGARd, and polyethylene diet (PEd)) and post-rearing PEd with indicated significant chemical shifts for alkene bonds (**A**), and for alcoholic or unsaturated allyl and carbonyl moieties (**B**)

To support the effective oxidation of plastics, as predicted by metagenomic analysis, chemical modifications of PE in post-rearing substrate (post-rearing PEd) were studied by ^1^H NMR analysis (Additional file 1: Figure S5). Indeed, using this technique, we could characterize PE degradation through oxidation in culture and within the insect gut [34, 104, 110, 111]. PEd, AGARd, and post-rearing AGARd served as controls (Additional file 1: Figure S5; Fig. 6). Peaks resonating at around 5.3 ppm arose from the resonance of protons in alkene compounds and those within 0.9–2.9 ppm indicated the occurrence of aliphatic molecules [34, 112]. Although both the ^1^H NMR spectra of PEd and post-rearing PEd generally showed similar profiles, some clear differences were observed (Fig. 6). Indeed, post-rearing PEd spectra showed a new peak at around 5.4 ppm in a region associated with alkene bonds (C = C − H), absent in PEd (Fig. 6A). Similarly, the peaks around 2.62–2.48 ppm were only observed in post-rearing PEd and can be attributed to the resonance of protons in alcohols (C − OH) or to those bound to alfa carbon atoms (i.e., in terminal position) in unsaturated allyl (HCαH = C) and carbonyl (HCαC = O) moieties (Fig. 6B) [25, 113]. These peaks were not observed in spectra of post-rearing AGARd, indicating that they did not result from larvae contamination (e.g., frass and exuviae) or the extraction method. Overall, ^1^H NMR analysis revealed that PE was subjected to oxidation, which is the key step in biodegrading PE and petroleum-derived polymers in general [103, 114].

### Genome reconstruction highlights the presence of several unknown species in BSF larval midgut

**Fig. 7:**
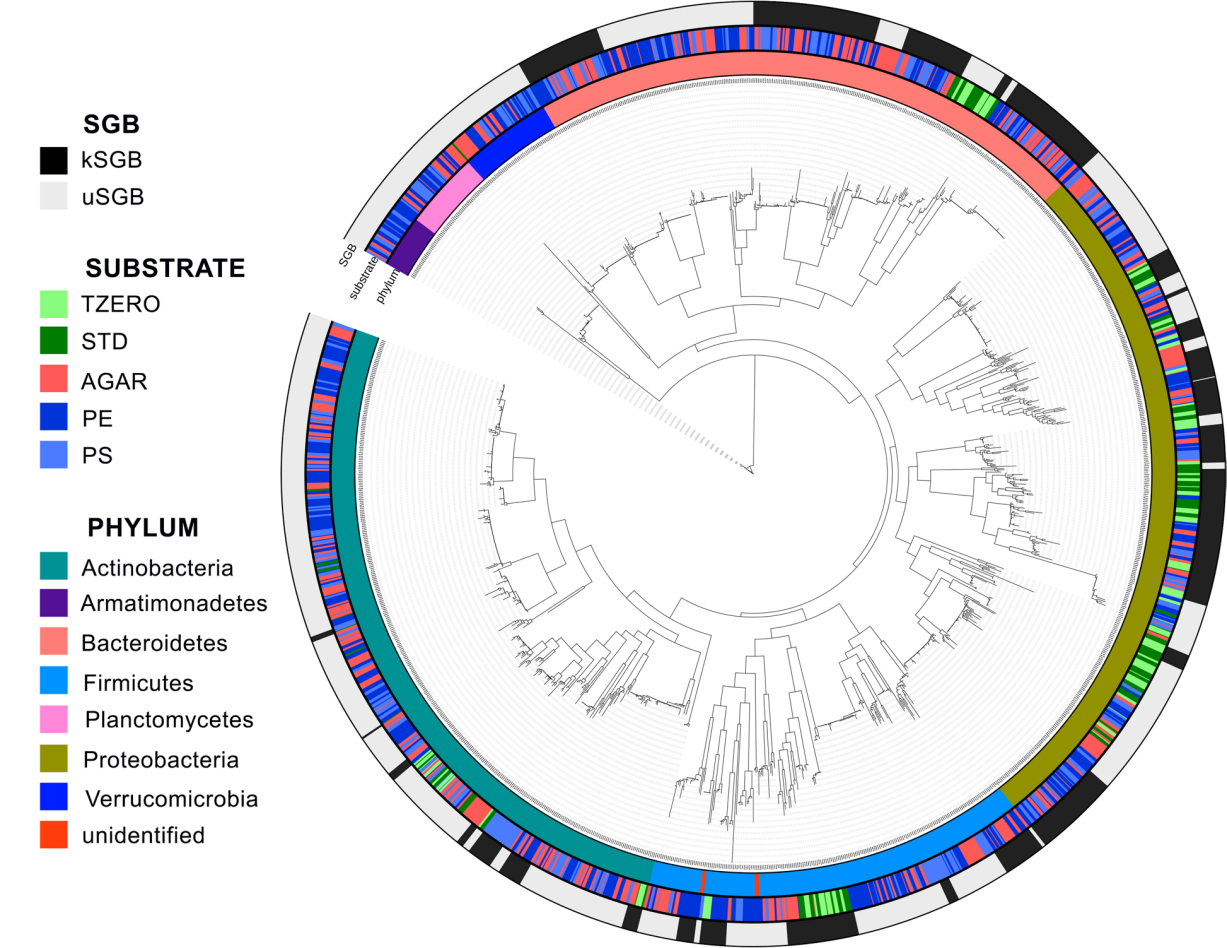
Phylogenetic tree of the 1547 MAGs reconstructed from metagenomes of BSF larval midgut and divided into 136 species-level genome bins (SGBs). From inside to outside, the rings are colored according to phylum-level taxonomy; rearing substrate (*T*_zero_, standard (STD), AGAR, polyethylene (PE), and polystyrene (PS) samples); presence of a reference genome in the SGB (known, kSGB or unknown, uSGB)

In all, 1547 MAGs were reconstructed by employing a metagenome-assembly approach, passing the thresholds for being defined as medium quality (MQ) according to recent guidelines (completeness > 50% and contamination < 5%; [115]). The 1547 genomes were clustered into species-level genome bins (SGBs), using an all-versus-all genetic distance quantification followed by clustering and identification of genome bins spanning a 5% genetic diversity. With this approach, we obtained 136 SGBs. Among them, 76 SGBs (including a total of 957 MAGs, about 62%) displayed > 5% genetic distance from the closest reference genome and therefore they represented species without any publicly available genomes from isolate sequencing or previous metagenomic assemblies (unknown SGBs, uSGBs). Only 590 (about 38%) of the MAGs represented at least partially known SGBs (kSGBs) that included one or more genomes in public databases (Fig. 7; Additional file 1: Figure S6).

The phylum-level taxonomy could be assigned to almost all the MAGs. Although the overall taxonomic composition at phylum level reflected that obtained by metagenome sequencing (Fig. 3A, B), we identified 27 MAGs belonging to Armatimonadetes (uSGB78), which were only present in AGAR, PE, and PS samples (Fig. 3C). In addition, one SGB (uSGB17) was assigned to Verrucomicrobia phylum. These two phyla were not detected using the metagenome mapping-based approach (Fig. 3B), highlighting the scarce availability of reference genomes from insect gut in public repositories and the importance of this approach to detect uncharacterized species.

Most of the SGBs were exclusive of BSF larvae grown on standard diet (*T*_zero_ and STD samples; e.g., kSGB1302, kSGB1308, and uSGB1313) or AGAR, PE, and PS samples (e.g., kSGB3, kSGB6, and uSGB19), and only few of them contained MAGs binned from all the groups (e.g., uSGB7, kSGB10, uSGB14; Additional file 4: Table S3), highlighting the strong selection occurring at species level in response to the diet. In some cases, the SGB was exclusively found in PS and AGAR (e.g., uSGB12, identified as a Bacteroidetes species), or in PE and AGAR samples (e.g., uSGB77, identified as *Brevundimonas* sp.), while kSGB445 (*Gordonia rubripertincta*) and uSGB687 (unidentified Actinobacteria) were found only in PE or in PE and PS samples, respectively (Additional file 4: Table S3).

**Fig. 8:**
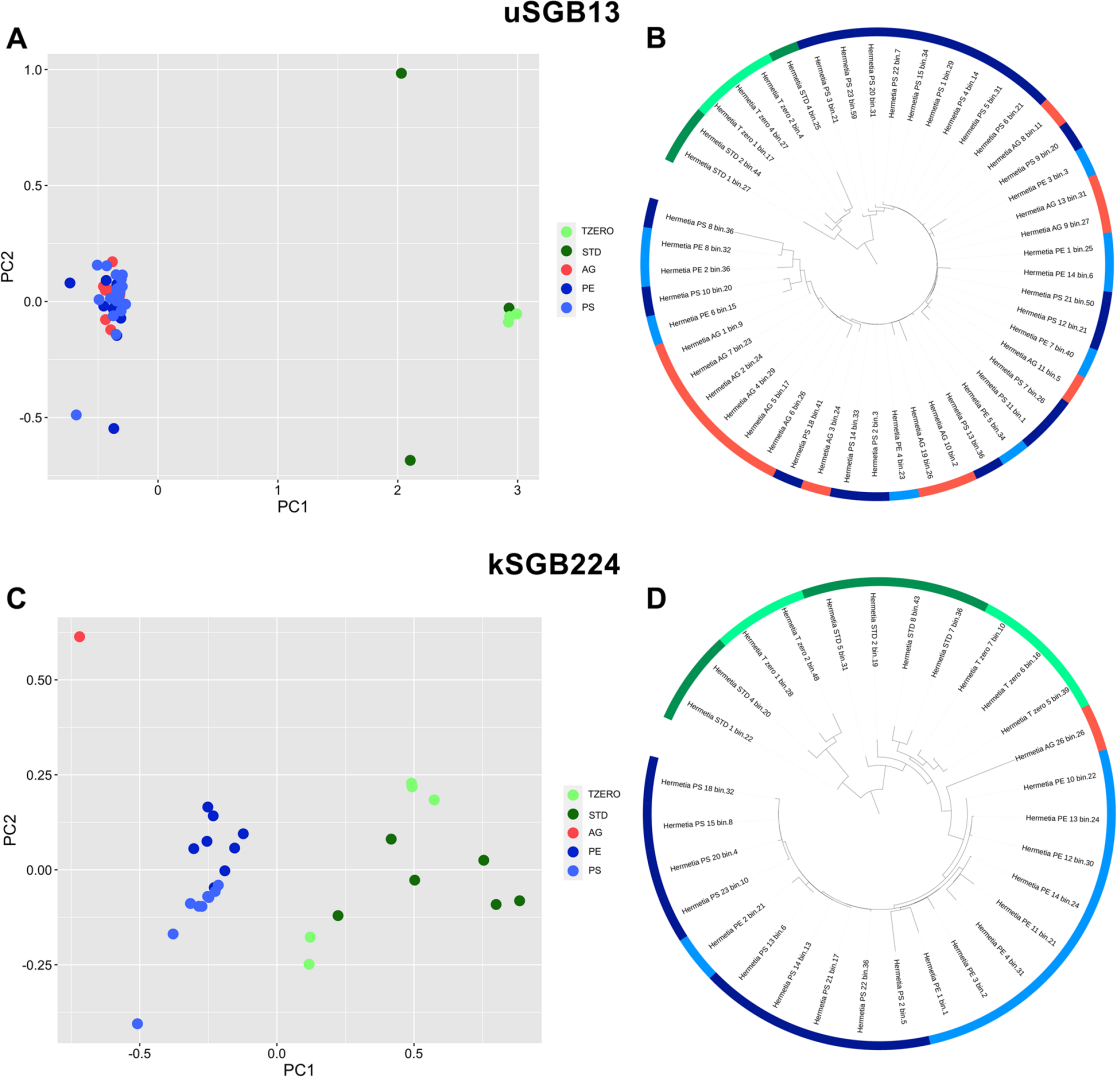
Selection from species-level genome bins (SGBs) at subspecies level occurs in response to diet. MAGs belonging to uSGB13 (unknown species from *Actinomycetaceae* family; **A**, **B**) and kSGB224 (*Sphingobacterium thalpophilum*; **C**, **D**) cluster according to the larvae diet in classical multidimensional scaling (MDS) carried out on ANI genetic distance matrix (**A**, **C**) and in phylogenetic trees (**B**, **D)**. Colors indicate the sample type (*T*_zero_, standard (STD), AGAR, polyethylene (PE), and polystyrene (PS))

Finally, evidence of a selection at subspecies level in response to diet was found. Based on prediction strength > 0.8 and PAM clustering, uSGB13 (unknown species from *Actinomycetaceae* family) and kSGB224 (*Sphingobacterium thalpophilum*) genomes could be divided into at least two subspecies each. Interestingly, one of the subspecies in uSGB13 was exclusive of larvae grown on STDd, while the second was found in AGAR, PE, and PS samples (Fig. 8A, B). In contrast, MAGs from kSGB224 were not recovered in AGAR (except for one sample), but a subspecies exclusive of PE and PS was identified, highlighting a selection driven by plastic-containing diet (Fig. 8C, D).

## Discussion

Several bacterial taxa with the potential ability to degrade petroleum-derived compounds, such as oils and plastics, have been related to the presence of these molecules in the environment where these microorganisms grow (e.g., landfill, soil, water, and insect gut). However, their ability to effectively degrade these recalcitrant molecules has only been shown for a few of them [13, 106, 116]. In recent years, insects (i.e., caterpillars and beetle larvae) have gained attention as plastic degraders thanks to their gut microbiota and have been proposed as a solution for developing innovative strategies for managing plastic waste [17, 106, 116]. Analyzing the structure of microbial communities exposed to PE and PS plastics constitutes the starting point for evaluating their biodegradation potential, while deciphering the underlying mechanisms is pivotal and has hardly been explored [5, 12, 13]. Recent work attempted to depict the gut microbiome in an insect reared on plastics; however, only a limited number of bacterial DNA sequences were obtained [117].

Here we used for the first time a saprophagous fly larva as a model (i.e., BSF larvae, the most widely used insect in bioconversion processes) to estimate the potential of its microbiome in plastic degradation. The BSF larval microbiome may represent a source of enzymes with novel and important functions and with industrial value thanks to some peculiarities of this insect. Indeed, as its midgut luminal pH ranges from 2 to 8.5, and as larvae have a gregarious habit that increases the temperature of the rearing substrate, their digestive enzymes show an optimal activity at about 45 °C [42, 118].

Growth on plastic-based substrates profoundly changed the composition of BSF larval microbiota, which is associated with a change in the surface of PE and PS particles: these particles appeared rough and cracked compared with the controls. In particular, the microbiota of larvae reared on PE and PS plastics was characterized by a significant increase in Actinobacteria compared with the control diet. It is worth pointing out that about half of the bacterial species that have been identified as *n*-alkane degraders so far are Actinobacteria [85]. In addition, we demonstrated that alkane hydroxylase and monooxygenase genes were significantly enriched in the microbial community of PE- and PS-fed BSF larvae, as were multicopper oxidase and laccase genes. This evidence supports the importance of oxidation reactions in plastic degradation and, as these enzymes are especially active on aromatic compounds, their presence may be critical for oxidizing PS aromatic rings. Plastics with a C–C backbone (e.g., PE and PS) are highly stable; therefore, oxidation represents the key, initial step for their further biodegradation [12, 17, 106, 109]. Bacterial oxidation of complex synthetic polymers is mediated by enzymes that are exploited to catalyze the breakdown of natural polymers with a similar structure (as hydrocarbons and lignin): interestingly, only a limited number of enzymes are responsible for this activity, and they are well conserved among different microbes [12, 17, 106, 109]. In particular, PE and PS degradation has been reported for microbial enzymes that are responsible for the oxidation and degradation of *n*-alkanes (i.e., alkane hydroxylases as alkane monooxygenases) [85, 119, 120], and lignin (class II peroxidases as lignin, manganese, versatile, and DyP-type peroxidases, and multicopper oxidases as laccases) [12, 86, 121–124].

Interestingly, although most multicopper oxidases with biotechnological potential are fungal in origin, from Actinobacteria have been isolated some of the few bacterial counterparts [125–127], as the laccase isolated from the actinomycete *Rhodococcus ruber* that has been reported to degrade PE [128]. The enrichment of multicopper oxidase and DyP-type sequences in the bacterial community of plastic-exposed BSF larvae is of extreme interest from a biotechnological point of view as these enzymes are increasingly finding application in biodegradation, bioremediation, and discoloration processes [121, 126, 127, 129]. It is worth mentioning that bacterial laccases are more interesting from the perspective of application than their fungal counterparts as they show a higher tolerance to a wider range of temperature and pH, a broader range of substrate specificity, shorter generation times, and easier manipulation for cloning and expression in a host [121, 126, 127, 129]. Unfortunately, though, bacterial laccases, peroxidases, and multicopper oxidases are less common than fungal ones and, in this respect, BSF larvae may help to improve the enzymes toolkit with biotechnological potential [12, 86, 121–123, 126, 127, 129].

In the present work, the changes in the genetic pool of the gut microbial community exposed to PE and PS strongly indicate that oxidative activity plays a key role in microbiome adaptation to plastics. Importantly, the functions predicted by microbiome analysis were corroborated by ^1^H NMR, which unequivocally showed changes in PE chemical features, i.e., the presence of hydroxylic and carbonylic groups absent in controls. This evidence was also supported also by alterations of the surface of the plastics. In addition to identifying enzymes able to degrade complex polymers and pollutants, gut microbiota of BSF larvae reared on plastics (alone or in combination) may thus represent a starting point for developing bacterial degrading consortia. The sharp increase in previously uncharacterized species in such microbiota points to the high potential for isolating bacterial species and strains which have peculiar and useful features. Indeed, MAG reconstruction highlighted that about 50% of the microbial community of the BSF larval gut is characterized by unknown species and that this proportion increases up to 70% when reared on plastic-containing substrates. Our results suggest that a strain-level selection may also occur in response to plastic-based diets, demonstrating the importance of selecting not only species but appropriate strains for plastic-degrading consortia.

Consortia isolated from marine water, soil, and landfills exhibited higher biodegradation activity on plastics such as PE, PS, and PET than did single species [130–133]. Moreover, as biodegradation of PE and PS mainly proceeds by oxidation catalyzed by different enzymes, it should be more efficient to convert them into monomers species with complementary metabolisms can cooperate. Recalcitrant polymers can be effectively oxidized by a microbial consortium with the cooperation of different enzymatic activities [12, 133] or by different enzymes produced by a single bacterium, as shown in a recent transcriptomic analysis where transcription of genes encoding both laccase-like and alkane monooxygenase enzymes increased upon exposure of a *Rhodococcus* sp. strain to PE [134].

## Conclusions

We have demonstrated that the gut microbial community of BSF larvae reared on PE and PS is characterized by high taxonomic diversity and genomic heterogeneity, suggesting that a network of mutualistic, synergistic, and competitive interactions is established both at the intraspecies and interspecies level. Thus, the BSF larval gut microbiota represents an optimal ecological niche for isolating enzymes and microbial strains with optimized plastic-degrading ability. Importantly, unraveling microbial genomes in the gut of the highly polyphagous BSF larvae reared on substrates of interest (as complex natural and synthetic polymers) may reveal new functions within the “unculturable” community and enable us to isolate enzymes for novel and bio-based industrial applications.

### Supplementary Information

**Additional file 1: Figure S1.** Growth and development of BSF larvae reared on different substrates, **Figure S2.** SEM analysis, **Figure S3.** Heatplot of sample clustering based on *16S rRNA* gene sequencing, **Figure S4.** Alpha-diversity indices of midgut microbiome, **Figure S5.** ^1^H NMR spectra, **Figure S6.** Relative abundance of known and unknown SGB.

**Additional file 2: Table S1.** Number of reads/samples, assembly results and number of predicted genes from *Hermetia illucens* larvae midgut metagenomes analysed.

**Additional file 3: Table S2.** Gene name and NCBI accession numbers of the sequences used to build the custom database used in this study.

**Additional file 4: Table S3.** List of the SGB from *Hermetia illucens* larvae midgut and MAGs reconstructed in this study.

## Data Availability

The raw sequence reads generated in this study have been deposited in the Sequence Read Archive (SRA) of the NCBI under accession numbers PRJNA864640 (shotgun metagenomes) and PRJNA873906 (*16S rRNA* sequences). Other raw data are available in Additional files.
