# Supplementary material for: Plastics shape the black soldier fly larvae gut microbiome and select for biodegrading functions
Source: Microbiome. 2023 Sep 14;11:205. doi: 10.1186/s40168-023-01649-0 (PMC10500907; doi:10.1186/s40168-023-01649-0)
Supplement: Supplementary file 2 — Additional file 1: Figure S1. Growth and development of BSF larvae reared on different substrates, Figure S2. SEM analysis, Figure S3. Heatplot of sample clustering based on 16S rRNA gene sequencing, Figure S4. Alpha-diversity indices of midgut microbiome, Figure S5. 1H NMR spectra, Figure S6. Relative abundance of known and unknown SGB. [file 40168_2023_1649_MOESM1_ESM.pdf]

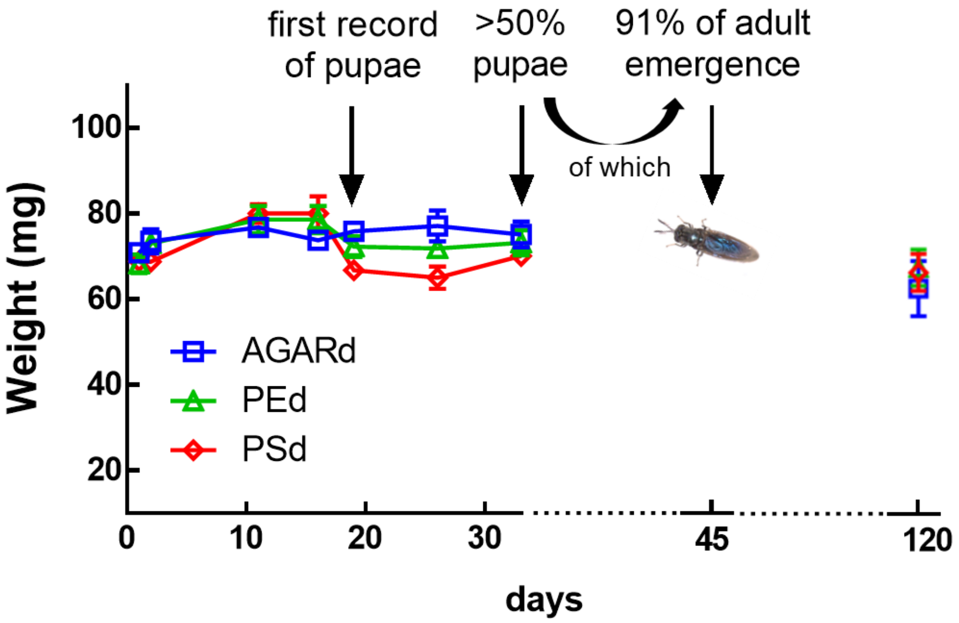

Fig. S1. Growth and development of BSF larvae reared on different substrates. After hatching, larvae were grown on standard diet (STDd) for 10 days and then moved to agar-based substrates containing PE and PS (PEd and PSd, respectively); a diet consisting of moistured agar (AGARd) served as control. Day 0 on the X axis corresponds to the day in which the larvae were transferred to AGARd, PEd, and PSd. Larval weight was recorded every 3-5 days until 50% of the larvae reached the pupal stage. The emergence of adults from recovered pupae and the weight of the larvae that survived, but did not pupate, after 4 months were also recorded.

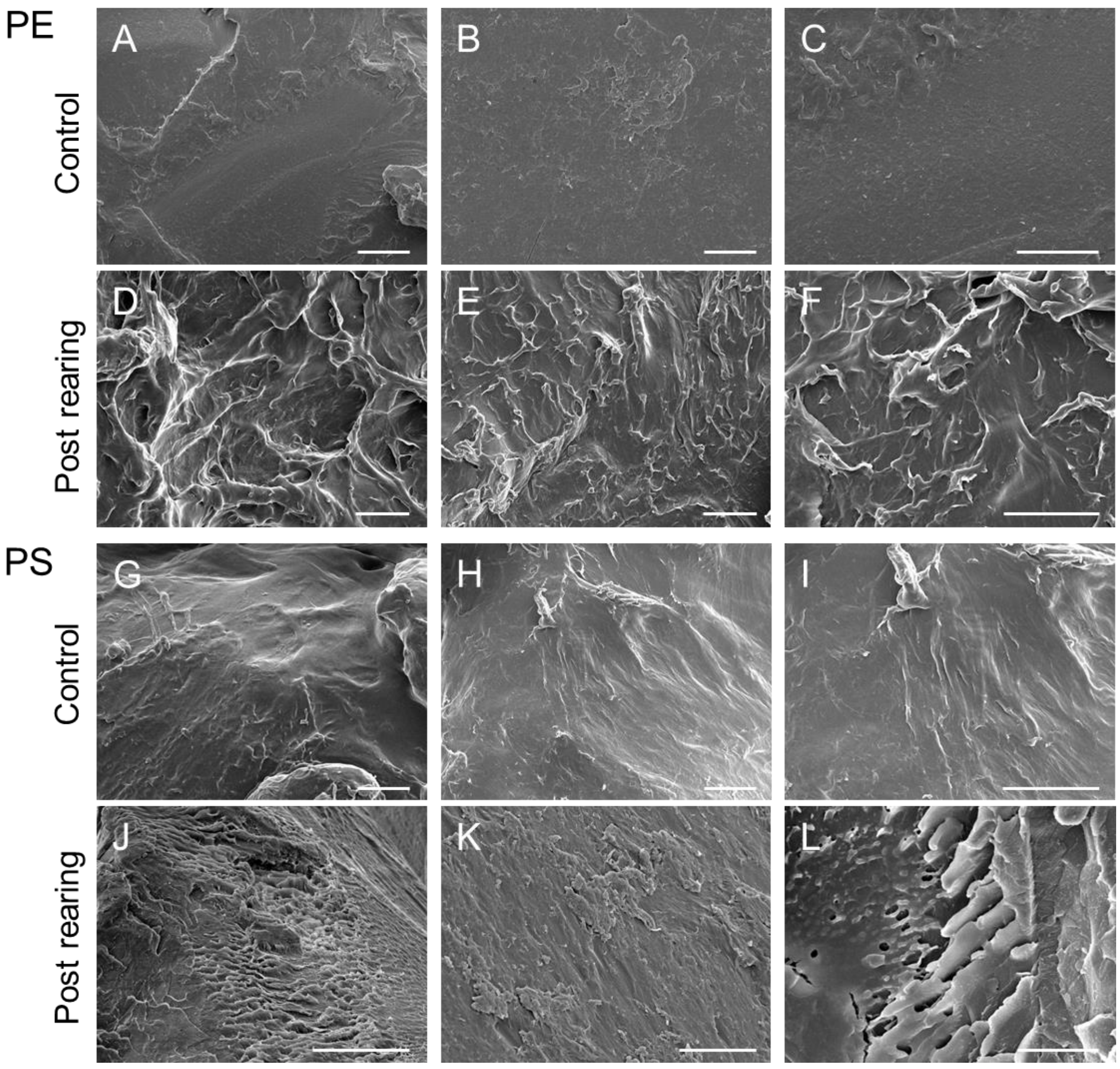

Fig. S2. SEM analysis. Morphology of plastics in the rearing substrate without the larvae (control) (A-C; G-I) and after rearing the larvae for 2 weeks (post rearing) (D-F; J-L). Bars: 20 µm (A, B, D-H, J-L), 10 µm (C, L).

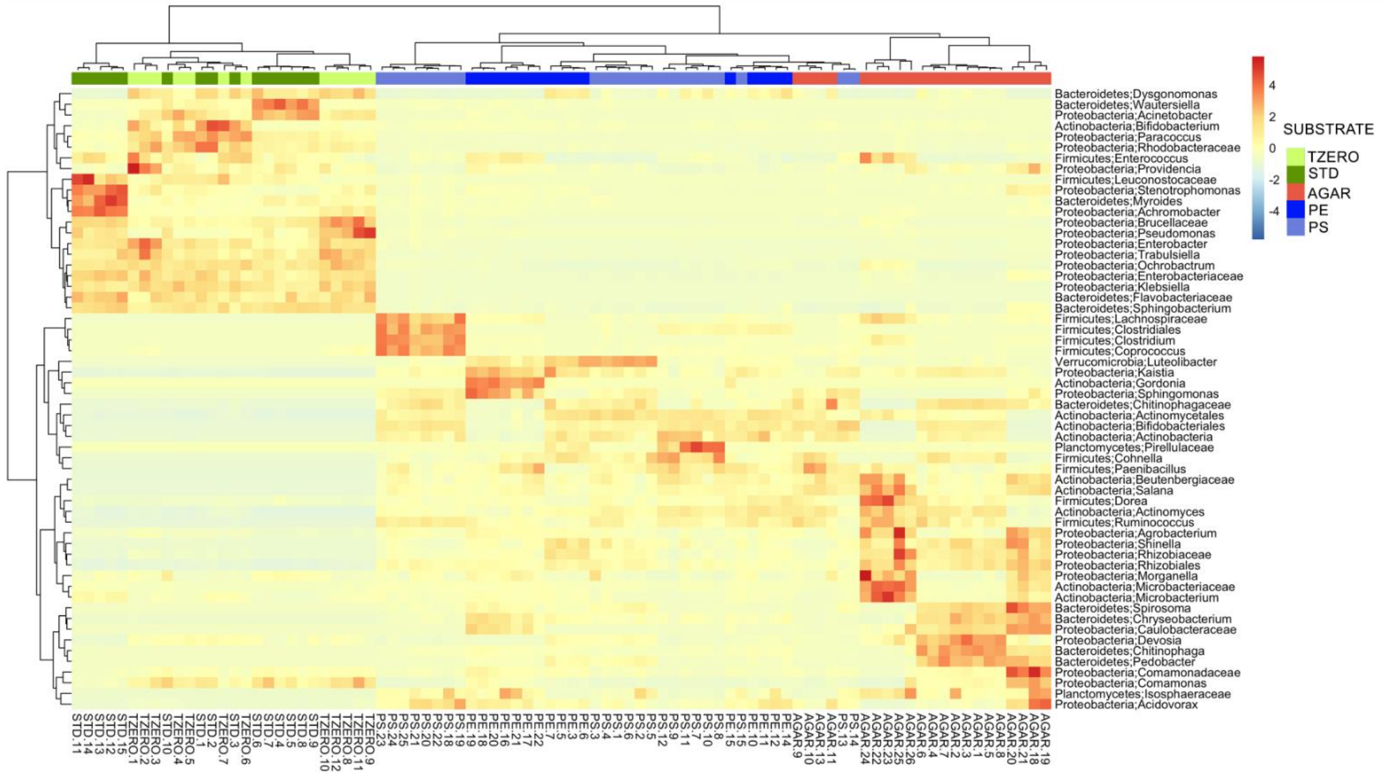

Fig. S3. Heatplot showing sample clustering based on gut microbiota composition. Only taxa with abundance > 0.5% in at least 5 samples are reported. Taxa are collapsed at genus level or above when genus identification was not achieved. Sample hierarchical Ward-linkage clustering is based on the Spearman's correlation coefficient of the taxa abundance. Column bar is coloured according to sample type (Tzero, STD, AGAR, PE, PS). The colour scale represents the scaled abundance of each variable, denoted as Z-score, with red indicating high abundance and blue indicating low abundance.

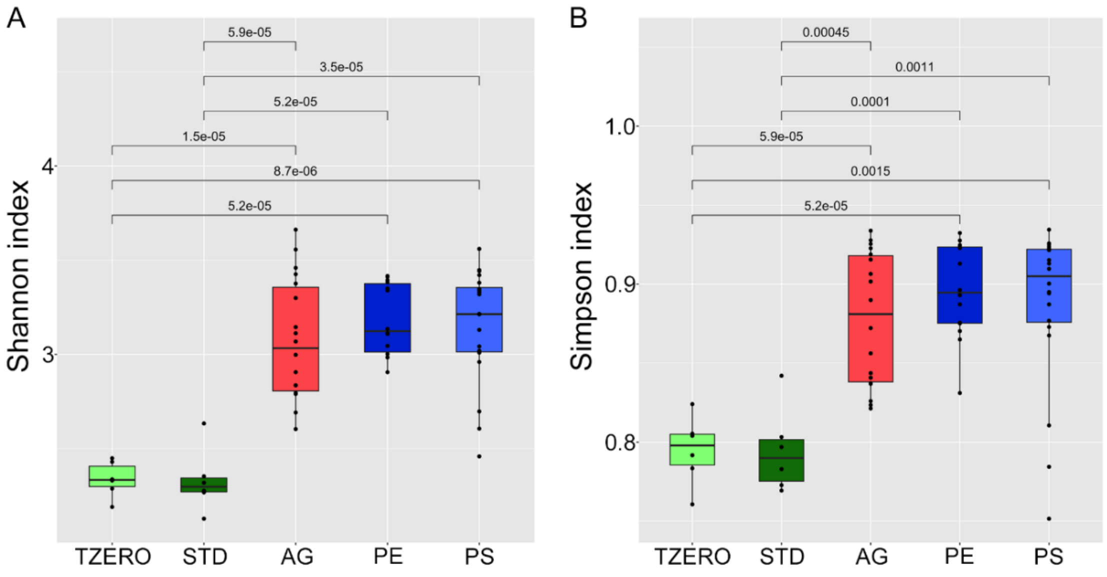

Fig. S4. Boxplots showing alpha-diversity indices of the midgut microbiome of T_{zero}, STD, AGAR, PE, and PS samples. Shannon (A) and Simpson (B) indexes were calculated on mOTU species-level table. Significance was determined by pairwise Wilcoxon tests. Only significant p-values ($p < 0.05$) are reported.

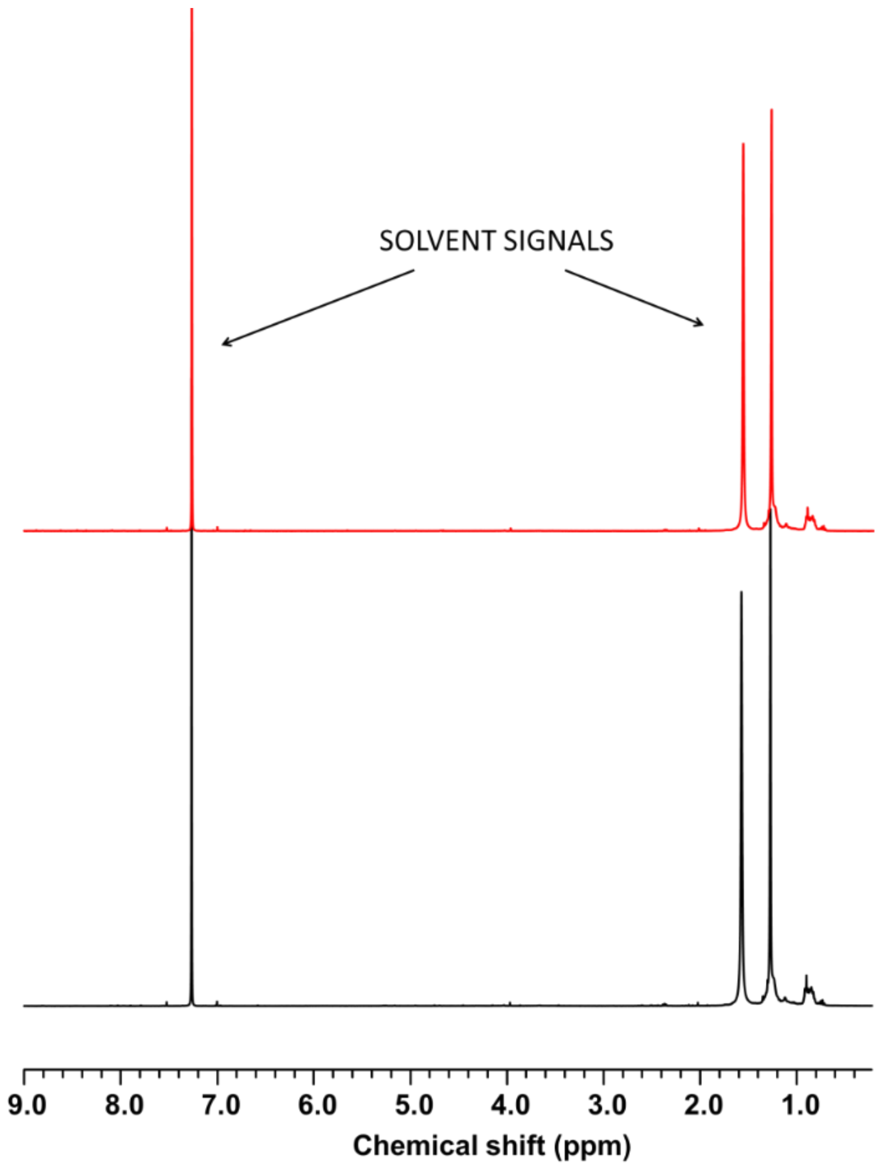

Fig. S5. Proton nuclear magnetic resonance analysis. ^{1}H NMR spectra of the rearing substrate containing PE without the larvae (control, red line) and after rearing the larvae for 2 weeks (black line).

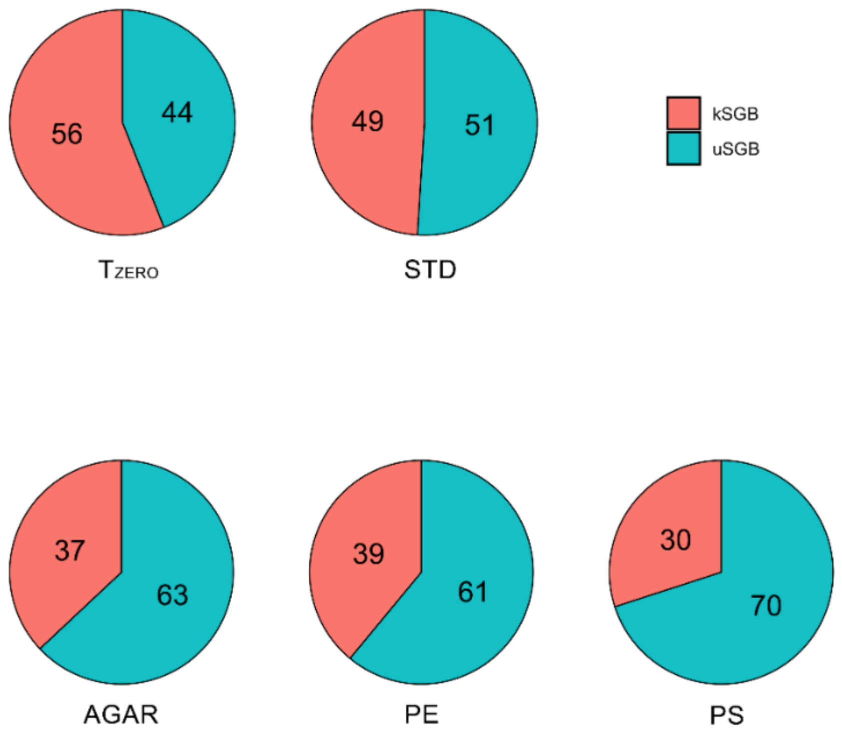

Fig. S6. Relative abundance of known and unknown SGB. Pie charts reporting the relative abundance (%) of SGBs identified at species level (kSGB; with < 5% genomic distance from a reference genome) or with > 5% genomic distance from the closest reference genome (uSGB) in T_{zero}, STD, AGAR, PE, and PS samples.
